# Italian Version of the Hospital Aggressive Behaviour Scale-Users: Initial Psychometric Evaluation among Hospital Healthcare Professionals

**DOI:** 10.3390/healthcare12171787

**Published:** 2024-09-06

**Authors:** Elena Cavallari, Ilaria Setti, Matteo Curcuruto, Cristina Gremita, Valentina Sommovigo

**Affiliations:** 1 Unit of Applied Psychology, Department of Brain and Behavioral Science, University of Pavia, 27100 Pavia, Italy; elena.cavallari01@universitadipavia.it (E.C.); ilaria.setti@unipv.it (I.S.); 2 Department of Human Sciences, European University of Rome, 00163 Rome, Italy; matteo.curcuruto@unier.it; 3 ATS Pavia, University of Pavia, 27100 Pavia, Italy; cristina_gremita@ats-pavia.it; 4 Department of Psychology, Sapienza University of Rome, 00163 Rome, Italy

**Keywords:** hospital healthcare professionals, workplace aggression from users, scale validation

## Abstract

Background: Healthcare professionals frequently encounter various forms of aggression, ranging from verbal abuse to physical assaults, which can compromise both their occupational well-being and patient-care quality. Despite its prevalence and serious consequences, workplace aggression is often underreported due to a lack of standardized assessment tools. This study aims to develop a valid Italian version of the Hospital Aggressive Behaviour Scale-Users. Methods: The scale’s structure was evaluated using exploratory (EFA) and confirmatory (CFA) factor analyses on two samples of healthcare professionals during and after the pandemic. Reliability, measurement invariance, and nomological validity were examined. Results: EFA revealed a two-factor structure comprising eight items (χ^2^ = 59.651, df = 13, *p* = 0.00; CFI = 0.98; TLI = 0.95; RMSEA = 0.07; SRMR = 0.02), distinguishing non-physical and physical aggression, and meeting all recommended criteria. CFA confirmed this structure, demonstrating good reliability and outperforming alternative models. The same factor structure was confirmed in standard (χ^2^ = 35.01, df = 19, *p* = 0.00; CFI = 0.99; TLI = 0.99; RMSEA = 0.03; SRMR = 0.02) and emergency (χ^2^ = 30.65, df = 19, *p* = 0.04; CFI = 0.98; TLI = 0.97; RMSEA = 0.06; SRMR = 0.04) contexts. Full residual invariance was found across job tenure groups. Aggression was positively associated with emotional exhaustion, psychological distance, psychosomatic symptoms, post-traumatic stress symptoms, and turnover intentions while negatively related to job satisfaction. Nurses and healthcare assistants reported higher levels of aggression than doctors. Conclusions: This study provides a reliable, context-specific instrument for documenting and analysing outsider aggression. The insights can inform targeted interventions, contributing to a healthier hospital environment.

## 1. Introduction

The National Institute for Occupational Safety and Health (NIOSH) defines workplace violence as “an act or threat of violence on a spectrum that ranges from verbal abuse to physical and even lethal assault towards persons at work or on duty” [1]. Workplace violence represents a significant risk factor for healthcare workers, who are among the most vulnerable professionals to both physical and verbal aggression [2]. Health personnel are five times more likely to experience workplace violence-related injuries than individuals in other professions [3], and workplace violence is four times more likely to occur in hospitals than in other settings [4]. In 2018, 73% of all nonfatal workplace violence-related injuries involved healthcare workers [3]. This high prevalence reveals only the visible part of the issue, as many cases are underreported [5]. Since the onset of the COVID-19 pandemic, the incidence of workplace violence has alarmingly increased globally [6,7]. For instance, in 2022 alone, over 1600 cases of aggression and violence against healthcare workers were reported to Italy’s National Institute for Insurance against Accidents at Work (INAIL), marking a notable rise from previous years [8].

Geographically, Northern Italy bears a disproportionate burden, with nearly 60% of these incidents occurring in regions like Lombardy, which is the setting of the present study [8]. This is likely due to its greater population density, industrial and economic significance, and extensive healthcare infrastructure [9]. The region’s high levels of urbanization and economic activity might contribute to more frequent reporting of incidents, making it more susceptible than less densely populated or economically active areas [10]. Additionally, Lombardy was among the regions hardest hit by COVID-19 during the initial wave, which severely strained the national healthcare system and its staff [11,12].

Workplace violence in healthcare encompasses a variety of forms, ranging from verbal abuse, such as yelling, snide comments, rude behaviour, ignoring, and humiliating actions, to more severe acts of physical violence [13]. The most common type of violence in healthcare settings is perpetrated by patients, families, or visitors [14]. According to a 2019 survey on healthcare-related crime, approximately 78% of aggravated assaults and 88% of all assaults in hospitals were committed by patients and family members [15].

### 1.1. Aggression from Users

Aggression from users, including patients and visitors, has a complex etiology influenced by various interconnected elements. Within the healthcare sector, organizational and systemic factors such as high-stress work environments, overcrowding, resource shortages, long waiting times, low-quality services, and insufficient information can impede the ability to meet patients’ and their relatives’ needs, potentially triggering aggressive reactions [8,15]. Certain personal characteristics may also increase the likelihood of some users exhibiting aggressive behaviour [16,17,18]. For instance, patients with mental disorders or cognitive impairments or those experiencing anger, anxiety, and frustration, combined with dissatisfaction with services, may exhibit aggressive behaviours [17,18].

Likewise, certain personal features may predispose some healthcare professionals to be more frequently targeted by user aggression. For instance, women healthcare professionals are more susceptible to verbal aggression, while men are more prone to physical violence [19]. Nurses, paramedics, healthcare workers in emergency departments, and those directly involved in patient care, such as in primary care or mental health, who work longer hours per week, are more likely to experience both physical and non-physical aggression [7,20,21].

User aggression can represent a potentially traumatic event in the workplace because it is characterized by its sudden occurrence during routine activities in environments typically perceived as safe (i.e., the workplace) [17]. Healthcare workers who are victims of workplace aggression must return to these settings, which can intensify and prolong their stress responses over time [22]. Although only a minority of healthcare professionals develop severe and persistent post-traumatic stress disorder symptoms that meet diagnostic criteria, it is common for victims to experience one or more post-traumatic symptoms in the aftermath, impairing clinical performance [23,24,25]. Returning to the workplace where the trauma occurred can trigger memories and emotions associated with the incident, potentially inducing flashbacks and intrusive thoughts of the aggression (i.e., re-experiencing symptoms), which can impair concentration and task completion [24,25,26]. Avoiding reminders of trauma may disrupt workplace relationships, leading to social isolation and reduced communication with colleagues and supervisors, as well as behaviours such as absenteeism [27] and turnover intentions, with an estimated annual turnover rate ranging from 15% to 36% due to workplace violence [28]. Sustained vigilance for potential threats and exaggerated emotional reactivity (i.e., hyperarousal symptoms) may generate mental fatigue, psychosomatic symptoms, and burnout [15,25,29,30,31]. Negative emotions can impact job satisfaction, while negative changes in cognition can diminish self-perception and confidence, hindering the ability to complete tasks effectively [15,24,25,29,30,31].

Due to the prevalence of patient aggression and its potentially harmful effects on occupational distress and work-related outcomes, it is crucial to accurately and comprehensively assess this phenomenon. By thoroughly evaluating the nature and extent of aggression in healthcare settings, targeted improvement and management strategies can be developed to protect the well-being of healthcare workers, ensure the quality of patient care, and maintain the overall stability of the healthcare system.

### 1.2. The Hospital Aggressive Behaviours Scale-Users

To date, researchers and key associations focused on worker protection and welfare have developed self-report surveys and questionnaires [32,33], in addition to objective measures of outsider aggression, such as incident reporting systems and occupational health reports, e.g., [34]. These tools effectively analyse healthcare workers’ experiences and identify potential hazards and factors that can trigger verbal or physical assaults. In addition to aggression-specific instruments, stress-related self-report measures have also been employed within the healthcare context to assess workplace stressors potentially associated with aggressive incidents, e.g., [35,36].

In the Italian healthcare context, many studies have utilized ad hoc instruments specifically developed by the authors to assess aggression from patients or users, e.g., [37,38]. However, these measures often lack validation and scientific rigor, making them unsuitable for generalization to other healthcare settings. Other studies used checklists such as the Violent Incident Form [39] or the Workplace Violence in the Health Sector Questionnaire [1,10,34,40,41,42]. Although these measures, typically using dichotomous yes/no responses, address various forms of mistreatment, they do not systematically capture the complexity of aggressive behaviours. A comprehensive tool that presents real-life scenarios occurring in the workplace and allows for the assessment of the frequency and severity of aggressive incidents provides a richer and more detailed understanding of the issue, rather than merely indicating the presence or absence of aggression. Additionally, such a tool helps identify specific triggers and patterns of aggression, which are crucial for developing targeted interventions.

Studies have also used the Italian version of Negative Acts Questionnaire-Revised (NAQ-R) [43,44], along with its abbreviated version validated by Balducci and colleagues [45], to assess workplace violence perpetrated by users within the Italian healthcare sector, e.g., [46]. However, this tool was developed to assess mobbing and was not specifically designed or validated for healthcare professionals, potentially making it less suitable for capturing the unique dynamics of patient-related aggression in healthcare settings. Other measures, such as the Modified Overt Aggression Scale (MOAS) [47] and the Nurses’ Observation Scale for In-Patient Evaluation (NOISE) [48], have been validated by Margari and colleagues [49], but they are specifically focused on psychiatric contexts. Therefore, a tailored instrument that directly addresses the specificities of hospital healthcare environments is necessary for a more accurate and relevant assessment.

The Hospital Aggressive Behaviour Scale-User (HABS-U) was developed and validated with a sample of over 1400 nurses from several public hospitals in Spain [50]. Exploratory and confirmatory factor analysis performed by Waschgler and colleagues [50] identified 10 items loading on two factors: non-physical and physical aggression. Participants responded to these items on a scale from 0 to 5 (0 = never; 5 = always), reflecting their work experiences with patients. To analyse criterion validation, Waschgler and colleagues [50] calculated correlations between the two factors and burnout, psychosomatic symptoms, and job satisfaction. They reported positive associations between both physical and non-physical aggression and both burnout and psychosomatic symptoms, as well as a negative correlation with job satisfaction [50]. Due to this, the HABS-U [50] represents a concise tool with good psychometric properties for evaluating healthcare workers’ experiences of user physical and nonphysical aggression. One dimension of the HABS-U [50] has been previously adopted in the Italian context. Sommovigo and colleagues [12] used it to assess verbal aggression from patients in a sample of healthcare professionals working in public hospitals, demonstrating good reliability. However, to our knowledge, an Italian version of the HABS-U [50] has not yet been validated.

To address this gap, this study aimed to investigate the factor structure of the HABS-U within the Italian context through exploratory and confirmatory factor analyses to create an initial Italian version of the scale. An additional objective was to determine whether the factorial structure would remain invariant when respondents were asked to focus on a specific source of aggression (i.e., patients) rather than on users in general (i.e., patients and visitors). We also aimed to analyse whether the factor structure of the HABS-U would be invariant across different job tenure groups (employees with low, moderate, or high experience in the current organization). This aspect is important as research on job tenure and workplace aggression is mixed, with some studies indicating that younger, less experienced healthcare workers are more frequently victimized [51], while others suggest that more experienced workers face higher levels of aggression due to increased responsibilities and patient interactions [21]. Furthermore, we explored the scale’s nomological validity and examined potential differences in perceptions of aggression from outsiders across gender, occupation, and years of experience.

We expect the Hospital Aggressive Behaviour Scale-Users to confirm a two-factor measurement model, even when respondents focus on a specific source of aggression and to demonstrate invariance across job tenure groups. We hypothesized that total scale scores would positively correlate with emotional exhaustion, psychological distance, psycho-somatic symptoms, posttraumatic stress symptoms, and turnover intentions while negatively correlating with job satisfaction. This approach not only aims to replicate the findings by Waschgler and colleagues [50], which confirmed that user aggression can lead to psychosomatic and burnout symptoms, as well as job dissatisfaction, but also seeks to extend their work by showing that such aggression can be experienced as a potentially traumatic event, triggering posttraumatic stress symptoms and turnover intentions.

Overall, this study aims to provide scholars and practitioners with a validated tool for measuring occurrences of non-physical and physical aggressive acts received by hospital healthcare professionals from users (i.e., patients and their visitors). Validating this instrument in Italian is crucial to ensure it is culturally and linguistically appropriate for Italian healthcare settings. A validated Italian version will enable more accurate assessments of patient aggression, providing a systematic tool for documenting incidents and ensuring that both non-physical and physical forms of user aggression are accurately captured. This is essential for understanding the true extent of the problem and identifying patterns and risk factors associated with different types of aggression. Additionally, data obtained from validated scales can inform targeted interventions and policies aimed at preventing and managing workplace violence. By identifying the most common sources and triggers of aggression, healthcare institutions can implement evidence-based strategies to mitigate these risks, such as staff training programs, environmental modifications, and comprehensive support systems for affected personnel.

## 2. Materials and Methods

### 2.1. Participants and Procedure

The present study was conducted in two distinct phases. The first phase (Study 1), conducted between October 2020 and February 2021, involved collecting data from healthcare professionals in contact with patients during the second COVID-19 wave at a single public hospital in Lombardy, Italy. This study was commissioned by the Medical Director and the Dean of Medicine. A protocol of understanding between the hospital and the University of Pavia, approved on 11 August 2020 (Protocol No. 372), formalized the agreement to conduct the study. Within this protocol, the hospital’s Ethical Review Board granted ethical approval for the research. From the outset, our goal was to use this dataset for comparative analysis. The medical director authorized the study and informed the staff about the research via email through the hospital’s intranet. Additionally, a coordinator and a researcher presented the research objectives to professionals during shift changes. After providing informed consent, 201 participants (response rate: 41.44%) completed anonymous self-report paper-and-pencil questionnaires. Of these, four were eliminated due to incomplete responses, and 28 due to being outliers. The cover sheet of the questionnaire informed participants about the study’s goals and assured them of the voluntary nature of their participation and the confidentiality of their responses. Once completed, the questionnaires were placed in cardboard boxes to ensure anonymity. There were no missing data for the scale items.

The second phase (Study 2), conducted between May and June 2024, expanded data collection to seven different hospital organizations within the same Northern Italian region. This phase also received separate ethical approval to ensure compliance with current ethical standards and protocols for multi-site research. It adhered to the ethical standards of the Italian National Psychological Association and was approved by the Ethical Committee of the University of Pavia. The researchers prepared a formal and detailed communication regarding the project’s objectives and operational methods. This communication was sent to the management of various departments and was then cascaded down to all employees through their direct supervisors. Participants were assured that their responses would remain anonymous, provided informed consent, and then completed the online survey, which took approximately 15 min to complete. A total of 1767 healthcare workers (response rate: 30.23%) completed the survey. We removed 119 cases for failing to complete at least 60% of the survey, reducing the sample size to 1648. An additional 206 cases were excluded after being identified as multivariate outliers, resulting in a final sample of 1442 respondents. The average percentage of missing values for continuous variables (scale items and variables included in nomological validity) ranged from 0% to 0.5%. The results of Little’s MCAR test were statistically non-significant (χ² = 1129.06, df = 1134, *p* = 0.54), indicating that the data were completely missing at random. Participants were randomly assigned to two distinct groups: one group, comprising 746 participants, was used for Exploratory Factor Analysis (EFA), while the other group, with 696 participants, was used for Confirmatory Factor Analysis (CFA1). This approach was designed to avoid overfitting and to independently validate the factor structure identified during the EFA. By utilizing separate samples for EFA and CFA, we aimed to enhance the robustness and generalizability of the factorial structure, a widely accepted method for enhancing the reliability of factor analyses [52,53,54,55]. Specifically, EFA was performed on 50% of the randomly assigned sample, and CFA was conducted on the remaining 50%. This methodology ensures that if the factorial structure identified in the EFA is confirmed with the CFA, the structure’s validity and reliability are strengthened [56]. Moreover, to examine the scale’s applicability in both standard and emergency contexts, its factorial structure was further validated using the sample collected during the initial phase, which occurred during an exceptional situation, namely the COVID-19 pandemic, providing an exemplary condition to test the scale robustness.

Most respondents in the EFA group were female (79.2%) and nurses (36.7%), with 75.3% in stable relationships and an average job tenure of 15.33 years (SD = 12.03). In the CFA1 group, most participants were female (76.4%) and nurses (34.5%), with 77.7% in stable relationships and an average job tenure of 15.86 years (SD = 12.07). Both groups reported similar average aggression scores (M = 0.65, SD = 0.65 for the EFA group; M = 0.70, SD = 0.68 for the CFA1 group; see Table 1). In the CFA2 group, most participants were female (76.4%) and nurses (34.5%), with 77.7% in stable relationships with an average job tenure of 15.24 years (SD = 12.19).

### 2.2. Measurements 

Aggression from patients and visitors was assessed using the 10-item Hospital Aggressive Behaviour Scale [50]. Participants reported the frequency of non-physical aggression (e.g., “Users get angry with me because of delay”) and physical aggression (e.g., “Users have shoved, shaken, or spit at me”) from outsiders on a five-point Likert scale (0 = never, 4 = daily).

Burnout was measured using three dimensions from the Italian version of the short Burnout Assessment Tool [57]: exhaustion (eight items, e.g., “At work, I feel mentally exhausted”; α = 0.94), mental distance (six items, e.g., “I struggle to find any enthusiasm for my work”; α = 0.82), and psycho-somatic symptoms (five items, e.g., “I suffer from palpitations or chest pains”; α = 0.89). Respondents indicated the frequency of each symptom on a five-point Likert scale (1 = never, 5 = always).

Post-traumatic stress symptoms were measured using the six-item Impact of Event-Revised (IES-R) scale [58]. Participants indicated the frequency of symptoms of intrusion (two items; e.g., “Other things kept making me think about it”), avoidance (two items; e.g., “I tried not to think about it”), and hyperarousal (two items; e.g., “I felt watchful or on guard” [59]), on a four-point scale (1 = never, 4 = often), experienced following the most recent episode of aggression. Following previous scholars [60], rather than using a three-dimensional solution, a global score (ranging from 0 to 24) was calculated by summing the scores of each subscale, providing an overall measure of post-traumatic stress symptoms. The IES-R scale demonstrated good internal consistency (α = 0.88). Participants were instructed to consider the most recent episode of verbal or physical aggression from patients or their visitors when answering questions about post-traumatic stress symptoms.

Job satisfaction was measured using a single item to assess overall satisfaction (e.g., “How satisfied have you been with your work?”) [61]. Responses were provided on a 10-point scale (from 0 = no satisfaction to 10 = satisfaction), where higher scores indicate greater job satisfaction. This single item has been used in previous studies focused on robberies [61].

Turnover intentions were assessed with a single item adapted from Knudsen and colleagues [62]. Participants rated their agreement with the statement, “I am seriously thinking about quitting my job”, using a seven-point Likert-type scale (1 = strongly disagree; 7 = strongly agree). This item was used in previous validation studies [62]. 

### 2.3. Translation

The HABS-U was translated following standard guidelines for translating questionnaires [63]. Initially, a native Italian-speaking researcher translated the items. To address potential issues with the translation, a bilingual expert panel (fluent in both English and Italian) reviewed the forward translation and suggested appropriate alternatives. An independent translator, who had not been involved in the initial translation, then translated all items back into English. Finally, native speakers of both Italian and English compared the back-translated version with the original scale, making further adjustments as needed.

### 2.4. Statistical Analyses

We utilized descriptive statistics to assess the distribution and reliability of the dataset before proceeding with further analysis using SPSS version 25 [64]. Multivariate outliers were identified using a significance level of *p* < 0.001 for Mahalanobis distance, and statistical assumptions were tested with the Kaiser–Meyer–Olkin measure and Bartlett’s test of sphericity. Mplus version 7 [65] was subsequently employed to conduct a parallel analysis to determine the appropriate number of factors to retain, a method known for its effectiveness in preventing over-extraction of factors [66]. The parallel analysis revealed that two observed eigenvalues exceeded the average of the expected eigenvalues, leading to a two-factor Exploratory Factor Analysis (EFA) on the EFA group. Due to skewness and kurtosis values indicating a non-normal distribution for three items, this analysis used maximum likelihood parameter estimates with robust standard errors (MLR) and a chi-square test statistic (where applicable) in Mplus, which is resistant to non-normality. We examined eigenvalues, communalities, and factor loadings for each item, along with item-total correlation coefficients and item discrimination indices to identify and remove poorly performing items.

A factor structure was established where all items exhibited loadings above 0.40 on their primary factor [67], with communalities surpassing 0.20 and item-total correlations exceeding 0.30 [55]. Following the guidelines provided by Howard [67] for making decisions in EFA, the factor analysis was repeated until a structure emerged in which all items (a) had loadings above 0.40 on their main factor, (b) had loadings below 0.30 on any other factors, and (c) showed a minimum difference of 0.20 between their primary and secondary factor loadings. A corresponding principal component analysis was then conducted in SPSS to confirm that the retained items explained a sufficient amount of the variance. Reliability measures, including Cronbach’s alpha, composite reliability (CR), and average variance extracted (AVE), were calculated using SPSS version 25 [68].

Next, in order to validate the factor structure derived from the EFA, a Confirmatory Factor Analysis (CFA) was conducted on two separate CFA groups using the MLR method in Mplus version 7. Consistent with previous research [53,54,55], model fit was evaluated using indices such as the Comparative Fit Index (CFI, values above 0.95 were considered satisfactory), Tucker–Lewis’s index (TLI, values above 0.95 were considered satisfactory), Root-mean-square Error of Approximation (RMSEA, values below 0.80 were considered acceptable), and Standardized Root Mean Square Residual (SRMR, values below 0.80 were considered acceptable). Additionally, to examine measurement invariance across groups with varying years of experience, we performed four Multigroup Confirmatory Factor Analyses (MGCFAs) using the ML method in Mplus 7. To determine statistical differences between models, the Satorra–Bentler scaled χ^2^ was calculated by subtracting the χ^2^ value of the baseline model from that of the nested comparison model [69].

Moreover, since χ^2^ values are sensitive to sample size, the differences in CFI [70] between the freely estimated model and the constrained model were used to evaluate the fit for the nested models. A CFI difference of 0.01 or less suggests between-group invariance of the CFA models [71]. The model fit was also assessed using RMSEA and CFI values. The scale’s nomological validity was confirmed by analysing the correlations between the overall scale score (and its components) with psycho–physical malaise and job satisfaction across the entire sample (including both the EFA and CFA1 groups). To achieve this, correlations were then computed on the total sample using: (a) Pearson’s r to assess the strength of association between continuous variables; (b) Spearman’s rho correlation coefficients to evaluate the strength of relationship between ordinal variables or between ordinal/continuous and dichotomous variables; (c) Kendall’s coefficients of rank correlations tau-sub to analyse the strength of association between continuous and ordinal variables. Effect sizes were interpreted according to Cohen’s guidelines [72]. These analyses were conducted using SPSS version 25. Lastly, independent sample *t*-tests and analyses of variance (ANOVAs) were conducted to identify differences in experienced aggression scores across groups based on gender, years of job tenure in the current position, and role. Cohen’s d values were calculated, and post-hoc Bonferroni tests were performed to explore these differences.

## 3. Results

### 3.1. Exploratory Factor Analysis

A total of 74 multivariate outliers were identified through Mahalanobis distance scores and consequently removed from the analysis. The skewness and kurtosis values revealed a non-normal distribution of the items, with skewness ranging from −0.77 to 3.04 and kurtosis ranging from −0.54 to −8.89. Bartlett’s test of sphericity was statistically significant (*p* < 0.001), and the Kaiser–Meyer–Olkin measure indicated satisfactory adequacy at 0.86. Results from the parallel analysis revealed that the factor in the original data produced an eigenvalue of 1.65, exceeding the average of the expected eigenvalues (eigenvalue = 1.13), thus supporting a two-factor structure. An initial five-factor EFA was conducted on the EFA group (*n* = 746) using the MLR method with Geomin rotation. However, two items in this solution did not fully meet the established criteria: the first item had a loading below 0.40 on its primary factor, while the last item loaded above 0.30 on an alternative factor and showed a difference of less than 0.20 between its primary and secondary factor loadings. As a result, these items were removed. The final two-factor solution in the EFA demonstrated a satisfactory fit (χ^2^ = 59.651, df = 13, *p* = 0.00; CFI = 0.98; TLI = 0.95; RMSEA = 0.07; RMSEA 95% CI = [0.05, 0.08]; SRMR = 0.02). All remaining eight item factor loadings met the recommended cut-off criteria outlined by Howard [67] (see Table 2).

Therefore, these findings supported a two-factor structure consisting of eight items, with factor loadings ranging from 0.65 to 0.92. The first factor, termed “non-physical aggression”, accounts for 51.45% of the variance, showed satisfactory internal reliabilities (α = 0.89; CR = 0.89) and included six items related to non-verbal actions by users (e.g., Users make ironic comments to me). The second item, labelled “physical aggression”, accounted for 19.75% of the variance, showed satisfactory internal reliabilities (α = 0.88; CR = 0.88), and comprised two items referring to physically aggressive actions by users (e.g., “Users have even shoved, shaken, or spat at me”). The inter-item correlation was 0.43, and the total scale demonstrated good internal consistency (α =0.86; CR = 0.93). Additionally, the item-total correlation averaged 0.69, and all items showed communalities ranging from 0.53 to 0.90. Ultimately, the factor solution accounted for 71.20% of the total variance.

### 3.2. Confirmatory Factor Analysis

Multivariate outliers were identified using the examination of Mahalanobis distance scores and were subsequently excluded from the analysis in the two CFA groups (i.e., 69 and 30 outliers for the first and second CFA groups, respectively). The items exhibited non-normal distribution, as indicated by skewness and kurtosis values (with indices ranging from 0.87 to 3.49 and kurtosis values from −0.17 to 11.81 for the first CFA group, and with skewness values ranging from 0.93 to 3.24 and kurtosis values ranging from −0.02 to 10.75 in the second CFA group). In both CFA groups, Bartlett’s test of sphericity was statistically significant (*p* < 0.001), and the Kaiser–Meyer–Olkin measure (i.e., 0.86 and 0.81) was appropriate. The two-factor model identified through the EFA was then validated on the two CFA groups (CFA_1_: *n* = 696; CFA_2_: *n* = 169) using the MLR method. This model demonstrated good fit indices in both CFA groups (CFA_1_: χ^2^ = 35.01, df = 19, *p* = 0.00; CFI = 0.99; TLI = 0.99; RMSEA = 0.03; RMSEA 95% CI = [0.01, 0.05]; SRMR = 0.02; CFA_2_: χ^2^ = 30.65, df = 19, *p* = 0.04; CFI = 0.98; TLI = 0.97; RMSEA = 0.06; RMSEA 95% CI = [0.01, 0.08]; SRMR = 0.04).

**Figure 1:**
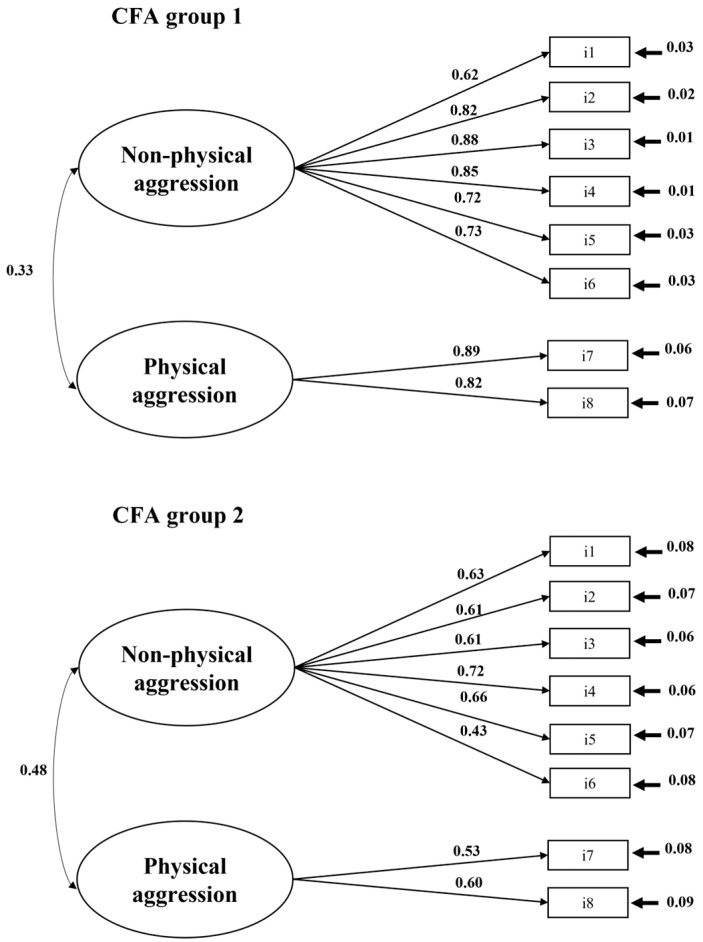
Standardized coefficients for the two-factor model across both CFA samples. Note. *p* < 0.001 for all coefficients.

Within each CFA group, we evaluated the fit of two competing models. The first model combined the two factors into a single factor, merging non-physical and physical aggression into one. The second model preserved the original factor structure, with the initial seven items loading onto the first factor and the three original items loading onto the second factor. The selected two-factor model with eight items outperformed all alternative models (see Table 3). In both CFA groups, all factors exhibited satisfactory composite reliabilities (CFA_1_: non-physical aggression: α = 0.89; CR = 0.90; physical aggression: α = 0.83; CR = 0.84; CFA_2_: non-physical aggression: α = 0.87; CR = 0.78; physical aggression: α = 0.95; CR = 0.50). Similarly, the total scale showed satisfactory reliabilities (CFA_1_: α = 0.87; CR = 0.93; CFA_2_: α = 0.95; CR = 0.82). In the first CFA group, the average inter-item correlation was 0.44, and the item-total correlation was 0.70, with item communalities ranging from 0.48 to 0.86. In the second CFA group, the average inter-item correlation was 0.47, and the item-total correlation was 0.73, with item communalities ranging from 0.53 to 0.95. In both CFA groups, the factor solution accounted for more than 70% of the total variance (i.e., CFA_1_: non-physical aggression: 52.70%; physical aggression: 18.60%; total: 71.31%; CFA_2_: non-physical aggression: 53.97%; physical aggression: 16.76%; total: 70.73%; see Figure 1 and Table 3).

### 3.3. Measurement Invariance

First, three CFAs were conducted separately for healthcare workers with fewer than 5 years of job tenure (χ^2^ = 52.60; df = 19; CFI = 0.97; TLI = 0.95; RMSEA = 0.07; RMSEA 95% CI = [0.05, 0.08]; SRMR = 0.03), those with 6 to 15 years (χ^2^ = 41.19; df = 19; CFI = 0.97; TLI = 0.96; RMSEA = 0.06; RMSEA 95% CI = [0.03, 0.09]; SRMR = 0.03), and those with more than 15 years of tenure (χ^2^ = 25.29; df = 19; CFI = 0.99; TLI = 0.99; RMSEA = 0.02; RMSEA 95% CI = [0.00, 0.05]; SRMR = 0.02). Next, we tested for measurement invariance across occupational groups through four MGCFAs (see Table 4).

The first was the configural model, which evaluates whether the number of factors and the pattern of indicator-factor loadings are equivalent across groups [73]. Results indicated adequate model fit, suggesting that the two-factor model and the factor pattern loadings were equivalent across job tenure groups. The second CFA evaluated the equality of factor loadings by constraining them to be equal across comparison groups. The difference in the χ^2^ statistic between the configural and metric invariance models was not statistically significant (Δχ^2^ = 15.54, Δdf = 16), and the difference in CFIs was below 0.01 (ΔCFI = 0.003), indicating that factor loadings were equivalent across job tenure groups. The third model tested scalar invariance, or the equivalence of item intercepts, by constraining the item intercepts to be equal across the groups while retaining the constraints from the metric invariance model. The difference in the χ² statistic between the metric and scalar invariance models was not statistically significant (Δχ² = 14.07, Δdf = 16), and the difference in CFIs was below 0.01 (ΔCFI = 0.001), indicating support for full scalar invariance. Next, the partial residual variance was tested by constraining all item residuals to be equivalent across the three groups while retaining the constraints from the scalar invariance model. The difference in the χ^2^ statistic between the scalar and residual invariance models was not statistically significant (Δχ^2^ = 9.24, Δdf = 7), and the difference in CFIs was below 0.01 (ΔCFI = 0.004). This suggests that a change in the latent variable produces the same effect on the score of the observed variables’ scores across job tenure groups, and that variations in all item scores are uniquely attributable to the latent variable. Therefore, observed scores at the scale means level (i.e., sums of item scores), their variances, and covariances can be compared, making it acceptable to compare means at the latent level across job tenure groups. Overall, these results indicated that HABS-U scores were comparable across job tenure groups.

### 3.4. Nomological Validity

Total and dimension scale scores were positively related to emotional exhaustion, psychological distance, psychosomatic symptoms, posttraumatic stress symptoms, and turnover intentions while negatively related to job satisfaction (see Table 5).

### 3.5. Results of Independent t-Test Analyses and Analyses of Variance

Further analyses were conducted to determine whether differences existed across gender, job tenure, and occupational groups regarding experienced aggression from outsiders (see Table 6). No statistically significant differences in aggression scores were found across gender and job tenure groups. Conversely, the ANOVAs (see Table 7) indicated statistically significant differences in total aggression (F_(4,1176)_ = 8.82, *p* < 0.001), non-physical (F_(4,1176)_ = 6.53, *p* < 0.001), and physical aggression (F_(4,1176)_ = 13.55, *p* < 0.001) across professional groups. Bonferroni’s post-hoc comparisons indicated that nurses reported experiencing more total aggression and non-physical aggression (total aggression: M = 0.77, SD = 0.69; non-physical aggression: M = 0.94, SD = 0.85) than doctors (total aggression: M = 0.57, SD = 0.48; non-physical aggression: M = 0.73, SD = 0.62) and laboratory technicians (total aggression: M = 0.50, SD = 0.64; non-physical aggression: M = 0.66, SD = 0.84). Moreover, nurses (M = 0.25, SD = 0.54) and healthcare assistants/auxiliary staff (M = 0.22, SD = 0.49) were more likely to report experiencing acts of physical aggression than doctors (M = 0.07, SD = 0.25) and laboratory technicians (M = 0.05, SD = 0.18).

## 4. Discussion

The present study sought to develop an initial Italian adaptation of the HABS-U [1] and evaluate its dimensional structure among hospital healthcare workers. Through exploratory factor analysis, the scale was shortened from 10 to 8 items, and the two-factor model proposed by Waschgler and colleagues [50] was validated.

The variation in the number of items can be attributed to the fact that the scale was administered not only to nurses but also to doctors, auxiliary personnel, laboratory technicians, social workers, and other healthcare professionals (e.g., physiotherapists, psychologists, obstetricians). As a result, the content of two items related to users becoming angry due to delays (i.e., “Users get angry with me because of delays”) and expressing their anger by damaging property (i.e., “Users show their anger at me by breaking doors, windows, or walls”) may not be universally applicable across all hospital healthcare workers due to differences in job roles and responsibilities. Additionally, Italian healthcare professionals might have interpreted the term “delay” as the extra time beyond the expected schedule rather than the total time users spent waiting for the required service irrespective of whether it matches the expected schedule (i.e., waiting times). This potential misinterpretation could have influenced their responses to items related to this concept on the scale. Furthermore, the correlations between aggression (total score and its dimensions) and burnout, post-traumatic stress symptoms, turnover intentions, and job satisfaction align with the hypothesized directions, supporting concurrent validity. Aggression from users, whether non-verbal or verbal, is positively correlated with emotional exhaustion, depersonalization, post-traumatic stress symptoms, psychosomatic symptoms, and turnover intentions, and negatively correlated with job satisfaction. These findings confirm that experiencing non-physical or physical aggression from users can have detrimental effects on the occupational well-being and job-related outcomes of hospital healthcare professionals.

Moreover, the current study found that nurses and healthcare assistants/auxiliary staff reported significantly higher levels of aggression compared to doctors and laboratory technicians. This result is understandable due to potential differences between these occupational groups in terms of job demands and exposure, which could influence their susceptibility to experiencing aggressions from users, as reported in previous studies [7,21,74]. The combination of direct patient interaction, physical proximity during caregiving (e.g., personal care and assistance with daily activities), and the nature of their caregiving roles (e.g., providing care during sensitive situations, such as administering treatments, or assisting with personal hygiene) may all contribute to nurses and healthcare assistants being at a higher risk of experiencing aggression from users compared to doctors and laboratory technicians [7,21,74]. For instance, the caregiving role of nurses and healthcare assistants involves addressing not only physical needs but also emotional and psychosocial aspects of patient care, such as managing user expectations and frustrations, which may contribute to situations where aggression arises [28,75]. Moreover, nurses and healthcare assistants are likely to be more visible and accessible to users compared to doctors and laboratory technicians, who may spend more time in offices or specialized areas. This increased visibility and accessibility might make nurses and healthcare assistants more susceptible to aggression from users.

Furthermore, the results supported full metric invariance across job tenure groups, indicating that hospital healthcare professionals generally interpret the content of these items similarly, regardless of their years of experience. In addition, the absence of statistically significant differences by job tenure might reflect the mixed findings in prior research on the relationship between job tenure and workplace violence [21,51]. On the one hand, experienced workers might become desensitized to or more skilled at managing aggressive incidents. On the other hand, newer employees might benefit from targeted training on handling patient aggression. This training could align their perceptions with those of their more experienced colleagues, who have developed refined coping strategies over time. Such training can equip newer staff with the skills and confidence needed to effectively manage and mitigate aggression, potentially leading to similar perceptions of aggression across different tenure groups. In many healthcare settings, workers with varying tenures work closely together, sharing experiences and support. This collaborative environment might result in a more uniform perception of aggression, as healthcare professionals could learn from each other and adopt similar coping strategies. Additionally, the repetitive and ongoing exposure to mistreatment may lead to habituation effects [76,77]. Over time, healthcare professionals might come to normalize experiences of aggression as part of their job, regardless of their tenure. This normalization process could contribute to a convergence in how aggression is perceived across different tenure groups, potentially masking statistically significant differences.

Our analysis validated the suitability of the items and demonstrated strong internal consistency for the scale. The reliability scores varied between 0.86 in the EFA group and 0.95 in the CFA2 group. The same two-factor structure was confirmed when respondents were asked to focus on aggressive behaviours from users in general (i.e., patients and their visitors) and, specifically, from patients alone. This indicates that the scale is effective for measuring aggression both broadly from users and specifically from patients. Furthermore, these results suggest that the HABS-U can effectively capture variations in perceptions of aggression depending on the source, providing flexibility in its application across different occupational groups within hospital healthcare settings, whether in standard or emergency situations.

Thus, the confirmed factorial structure of the HABS-U in both normal and exceptional circumstances, such as those during the COVID-19 pandemic, underscores its robustness across varying contexts. The pandemic period illustrates how unique environmental and relational factors can influence the occurrence of aggression in hospital settings. During this time, pervasive anxiety due to uncertainty, fear of infection, and high mortality rates heightened frustration and fear [78,79], leading to increased aggression from patients [12,80]. Despite the elevated anxiety levels post-pandemic, the reduction in immediate threats might have been expected to decrease stress-induced aggressive behaviours. However, our data indicate that aggression rates not only remained similar but increased post-pandemic. Strict visitation restrictions during the pandemic isolated patients and left families feeling helpless, which may have increased non-physical aggression, such as verbal confrontations, as a way of expressing dissatisfaction or fear. Although these restrictions limited aggression primarily to interactions with patients, the post-pandemic relaxation of visitation policies reintroduced visitors as potential sources of outsider aggression. Improved communication and support systems post-pandemic might have reduced some non-physical aggression, but increasing overall aggression rates suggests other intervening factors. The pandemic strained healthcare resources, leading to staff shortages, longer wait times, and overcrowded facilities, which further fuelled both physical and non-physical aggression due to perceived delays or inadequate care [12,81,82]. Even as healthcare systems stabilized post-pandemic, with better resource allocation and less overcrowding, the persistence and even rise in aggressive incidents highlight the lingering effects of the crisis on patient behaviour and the hospital environment. The mandatory use of personal protective equipment (PPE) during the pandemic, while essential for safety, created communication barriers and made healthcare workers appear less approachable, potentially increasing frustration and misunderstandings that contributed to aggression. As PPE use became less restrictive post-pandemic, these communication barriers likely decreased, which could have been expected to reduce instances of aggression related to perceived impersonal care. However, the continued or increased aggression rates post-pandemic suggest that the psychological and emotional impacts of the pandemic have had a lasting effect, necessitating ongoing attention to managing aggression in healthcare settings. Overall, our findings indicate that the Italian version of HABS-U is a user-friendly instrument for assessing users’ aggression towards healthcare workers in standard and extraordinary times.

The validation of the HABS-U advances our understanding of aggression in healthcare settings for several reasons. Unlike broader tools such as the NAQ-R [43,44], which focuses on mobbing rather than user-related aggression, or the MOAS [47] and the NOISE [48,49], which are designed specifically for psychiatric contexts, we provide a first validation of the HABS-U [50] with a focus on the hospital environment. This specificity addresses the unique dynamics of aggression experienced by healthcare workers from patients and users, an aspect not fully captured by existing tools. Many current tools, including ad hoc instruments and checklists used within the Italian context, either lack the depth and comprehensiveness required for a nuanced analysis or rely on dichotomous responses that fail to effectively measure frequency, severity, and triggers of aggressive behaviours. The HABS-U provides a detailed assessment that captures these critical aspects, offering a more thorough understanding of aggressive incidents in healthcare settings. Furthermore, existing tools often suffer from limited scientific validation or are unsuitable for generalization across various healthcare environments. The HABS-U addresses these shortcomings by providing a validated and contextually relevant measure, enhancing the ability to generalize findings across diverse hospital settings. By addressing these theoretical gaps, the HABS-U offers a more accurate assessment of aggressive behaviours in hospital environments, providing valuable insights that surpass the limitations of existing tools.

The study enhances the understanding of aggression in healthcare settings, supporting the development of targeted interventions on staff well-being by providing a reliable scale tailored for healthcare professionals working in Italian hospitals. The Italian validation of the HABS-U is crucial for advancing preventive and protective measures implementation as outlined in Recommendation No. 8 of the Italian Ministry of Health (2007) [83]. This aligns with the legislative focus on mitigating risk conditions and equipping healthcare workers to effectively manage incidents of aggression, as emphasized in Law No. 113 of 14 August 2020 [84]. Thus, the validated and comprehensive nature of the HABS-U enhances healthcare facilities’ ability to monitor and improve safety levels, supporting institutional objectives related to effective safety measures and surveillance tool usage.

By providing a precise understanding of the prevalence of both physical and non-physical aggression from users, the HABS-U facilitates a more effective allocation of resources, helping to prioritize training programs, security measures, and support services where they are most needed. The scale’s capacity to separately evaluate verbal and physical aggression allows the identification of specific areas requiring intervention. For instance, high scores in non-physical aggression may signal the need for training programs focused on communication skills, conflict resolution, and de-escalation strategies. Conversely, high levels of physical aggression may necessitate designing safer physical environments, enhancing security measures, and collaborating with security personnel to develop effective protocols for managing aggressive incidents. Additionally, analysing survey data across sociodemographic variables (e.g., gender, age) and organizational factors (e.g., department, role) can reveal variations in aggression levels, aiding in targeted intervention development. For instance, supervisors could organize regular team meetings to encourage staff to openly share their emotional experiences and relational challenges related to users in wards with high aggression rates [85]. This approach can help workers process their emotions and identify potential solutions. It is also crucial to conduct debriefing sessions immediately after aggressive incidents to enhance awareness of reactions and facilitate effective processing of the events. Establishing a listening centre or providing employee assistance programs and psychological counselling could further support staff well-being [22]. Additionally, implementing both individual and group mentoring sessions can help transfer skills to socio-demographic groups with greater needs, focusing on effective emotion management strategies during relational challenges with users. Regularly administrating the scale post-intervention can help monitor changes in employees’ perceptions of aggression, providing valuable data for policy development and protocols for managing aggressive behaviour revision. Overall, the validation of the HABS-U directly supports institutional guidelines by offering a robust, context-specific instrument for evaluating aggression. This tool aids in implementing effective preventive strategies, strengthens safety monitoring, supports targeted training initiatives, and fosters a deeper understanding of the challenges faced by healthcare professionals.

### Limitations and Suggestions for Future Research

The findings of this study should be interpreted with its limitations in mind. While the sample comprised healthcare professionals from eight hospitals in a single region of Northern Italy, the results may not apply to other regions or the wider population of Italian healthcare workers due to regional and demographic variations. Additionally, the sample was predominantly female and not evenly distributed across professional groups, limiting our ability to test measurement invariance across gender, and professional groups. Therefore, future research should aim to replicate these findings with larger, more representative samples that include a more balanced representation of socio-demographic characteristics among Italian healthcare workers. This would enable further investigation into the invariance of the HABS-U structure across different socio-demographic groups and enhance the robustness and generalizability of the model.

Additionally, the cross-sectional design and reliance on self-reported data prevent the establishment of causal relationships and may introduce limitations typical of such methodologies, such as potential social desirability bias. To address this, we followed questionnaire design guidelines suggested by Podsakoff and colleagues [86].

Future research could improve validity by gathering data from diverse sources (e.g., interviews, observations of actual behaviours) and employing a longitudinal design to evaluate the test-retest reliability of the Italian version of the HABS-U. Additionally, using objective measures (e.g., hospital incident reports) could complement self-reported data, providing a more holistic understanding of aggression in healthcare settings.

Moreover, due to the voluntary participation of respondents in this study, selection bias cannot be ruled out. Specifically, our data may be affected by the “healthy worker effect”, which could lead to an underestimation of burnout levels [22]. It is plausible that healthcare workers who participated in our study were sufficiently healthy to continue working, potentially resulting in their overrepresentation in the sample. On the other hand, healthcare workers suffering from burnout may have been absent or left the workforce due to health issues, resulting in their underrepresentation. To address this bias in future studies, incentives could be offered to encourage participation from all employees within a particular hospital, ensuring more comprehensive workforce representation.

Although tools such as the NAQ-R [43,44], NOISE [47], and MOAS [48,49] are available, we did not test discriminant validity in this study to keep the survey concise and to minimize issues related to response rates, ensuring effective data collection. Future research should investigate whether HABS-U evaluations are distinct from those of other measures, thus contributing further to its validation and practical application. Moreover, nomological validity was confirmed by showing that the scale’s correlations with other relevant variables (e.g., burnout, post-traumatic stress symptoms, turnover intentions, and job satisfaction) aligned with expectations. Future research should explore whether the HABS-U scale correlates with other constructs associated with outsider aggression. Introducing qualitative methods, such as interviews or focus groups, could also provide deeper insights into the nuanced experiences of healthcare workers with aggression, complementing quantitative findings.

## 5. Conclusions

This study provides initial validation of the Italian version of the Hospital Aggressive Behaviour Scale-User, offering a reliable tool for assessing the frequency and severity of incidents of outsider aggression. Recognizing and addressing such aggression through a validated scale is crucial for fostering a safe and supportive healthcare environment where hospital healthcare professionals can feel secure and healthy, which is an essential condition for ensuring high-quality care. In conclusion, the validated Italian version of the Hospital Aggressive Behaviour Scale-User is a valuable asset for healthcare organizations, potentially contributing to safer work environments, improved resource management, and enhanced healthcare delivery.

## Figures and Tables

**Table 1:** Descriptive statistics of EFA and CFA groups.

Variable	EFA Group (*n* = 746)	CFA_1_ Group (*n* = 696)	CFA_2_ Group (*n* = 169)
	%	%	%
Gender			
Female	79.2	76.4	76.9
Civil status			
In a stable relationship	75.3	77.7	77.8
Single	24.7	22.3	22.2
Role			
Doctor	15.6	16.4	19.4
Nurse	36.7	34.5	34.2
Healthcare assistants/auxiliary staff	8.7	7.8	9.0
Laboratory technician/radiologist	9.0	9.0	-
Social worker	1.0	1.0	-
Other healthcare professions (e.g., psychologist, obstetric, physiotherapist)	29.0	31.3	37.4
Work schedule			
Full time	91.8	89.7	100
Part time	8.2	10.3	-
	M (SD)	M (SD)	M (SD)
Job tenure	15.33 (12.03)	15.86 (12.07)	15.24 (12.19)
Average aggression	0.65 (0.65)	0.70 (0.68)	0.42 (0.53)
Average non-physical aggression	0.82 (0.82)	0.88 (0.86)	0.62 (0.67)
Average physical aggression	0.14 (0.39)	0.14 (0.42)	0.22 (0.56)

Note. EFA = Exploratory Factor Analysis; CFA_1_ = Confirmatory Factor Analysis post-COVID; CFA_2_ = Confirmatory Factor Analysis during COVID-19; % = Frequency; M = Mean; SD = Standard Deviation.

**Table 2:** EFA (*n* = 746): factor loadings and communalities of the selected items, explained variance and factor reliability.

Factor			
Items	Non-Physical Aggression	Physical Aggression	h^2^
Item 1	**0.65**	0.02	0.53
Item 2	**0.76**	0.00	0.66
Item 3	**0.82**	0.00	0.72
Item 4	**0.86**	−0.06	0.74
Item 5	**0.73**	−0.01	0.61
Item 6	**0.43**	0.06	0.64
Item 7	0.01	**0.92**	0.90
Item 8	−0.02	**0.85**	0.90
	Factor 1	Factor 2	Total
Explained variance (%)	51.45	19.75	71.20
Cronbach’s alpha	0.88	0.89	0.86
Composite reliability	0.89	0.88	0.93

Note. h^2^ = item communality. Factor loadings > |0.40| are in bold.

**Table 3:** Fit indices for the selected three-factor model and the alternative measurement models.

**Model CFA (*n* = 696)**	**χ^2^**	**df**	**CFI**	**TLI**	**RMSEA**	**RMSEA 90% CI**	**SRMR**	**AIC**	**BIC**
2-factor model ^c^	35.01	19	0.99	0.99	0.03	[0.01, 0.05]	0.02	11,177.39	11,291.02
2-factor model ^b^	271.13	34	0.92	0.89	0.10	[0.09, 0.11]	0.09	20,235.79	20,376.70
1-factor model ^a^	374.48	20	0.83	0.76	0.16	[0.15, 0.17]	0.10	11,643.16	11,752.24
**Model CFA (*n* = 169)**	**χ^2^**	**df**	**CFI**	**TLI**	**RMSEA**	**RMSEA 90% CI**	**SRMR**	**AIC**	**BIC**
2-factor model ^c^	30.65	19	0.98	0.97	0.06	[0.01, 0.08]	0.04	2377.79	2456.04
2-factor model ^b^	125.24	34	0.89	0.86	0.13	[0.10, 0.15]	0.07	3947.22	4044.25
1-factor model ^a^	216.98	20	0.64	0.49	0.24	[0.21, 0.27]	0.10	2615.41	2690.53
Cut-off			>0.095	>0.095	<0.08		<0.08		

Note. df = degree of freedom; RMSEA = Root Mean Square Error of Approximation; SRMR = Standardized Root Mean Square Residuals; CFI = Comparative Fit Index; TLI = Tucker–Lewis Index. ^a^ All selected eight items load on a single factor. ^b^ Original factorial structure model where the seven items of non-physical aggression load on the first factor, while the three items of physical aggression load on the second factor. ^c^ Selected model where six items of non-physical aggression load on the first factor, while the two items of physical aggression load on the second factor.

**Table 4:** MGCFA results for assessing measurement invariance across job tenure groups (*n* = 1442).

Model	χ^2^	df	Δχ^2^	Δdf	*p*	CFI	TLI	SRMR	RMSEA	90% CI RMSEA	ΔCFI
Model for less experienced professionals	52.60	19	-	-	-	0.972	0.95	0.03	0.07	[0.05, 0.08]	-
Model for middle-experienced professionals	41.19	19	-	-	-	0.973	0.96	0.03	0.06	[0.03, 0.09]	-
Model for more experienced professionals	25.29	19	-	-	-	0.996	0.99	0.02	0.02	[0.00, 0.05]	-
Configural invariance	118.36	57	-	-	-	0.983	0.97	0.05	0.03	[0.04, 0.06]	-
Metric invariance	126.34	73	15.64	16	0.48	0.986	0.98	0.05	0.04	[0.03, 0.05]	0.003
Scalar invariance	143.92	89	14.07	16	0.59	0.985	0.99	0.05	0.04	[0.03, 0.04]	0.001
Residual invariance	146.48	105	12.40	16	0.72	0.989	0.99	0.05	0.03	[0.02, 0. 04]	0.004

Note. df = degree of freedom; Δχ^2^ = difference in chi-square between models; RMSEA = Root Mean Square Error of Approximation; 90% CI RMSEA = 90% confidence interval RMSEA; CFI = Comparative Fit Index; ΔCFI = difference in CFI between models.

**Table 5:** Descriptive statistics and intercorrelations among the study’s variables in the total sample (*n* = 1442).

	M	SD	1	2	3	4	5	6	7	8	9	10
1. Total scale	0.67	0.66	0.86									
2. Non-physical aggression	0.85	0.84	0.99 **^a^	**0.89**								
3. Physical aggression	0.14	0.40	0.42 **^a^	0.28 **^a^	**0.85**							
4. Emotional exhaustion	2.41	0.93	0.36 **^a^	0.36 **^a^	0.14 **^a^	**0.94**						
5. Psychological distance	1.71	0.74	0.32 **^a^	0.33 **^a^	0.07 **^a^	0.59 **^a^	**0.82**					
6. Psychosomatic symptoms	2.11	0.83	0.27 **^a^	0.27 **^a^	0.09 **^a^	0.56 **^a^	0.42 **^a^	**0.89**				
7. Posttraumatic stress symptoms	1.05	0.90	0.42 **^a^	0.42 **^a^	0.14 **^a^	0.51 **^a^	0.44 **^a^	0.44 **^a^	**0.88**			
8. Job satisfaction	6.62	2.24	−0.22 **^a^	−0.22 **^a^	−0.09 **^a^	−0.47 **^a^	−0.58 **^a^	−0.33 **^a^	−0.24 **^a^	-		
9. Turnover	2.50	1.87	0.19 **^a^	0.19 **^a^	0.09 **^a^	0.45 **^a^	0.50 **^a^	0.32 **^a^	0.23 **^a^	−0.51 **^a^	-	
10. Sex	-	-	0.05 *^b^	0.05 ^b^	0.04 ^b^	0.08 **^b^	0.00 ^b^	0.24 **^b^	0.05 *^b^	−0.08 **^b^	0.03 ^b^	-
11. Job tenure	15.58	12.05	0.04 ^b^	−0.03 ^c^	−0.01 ^c^	0.05 **^c^	0.06 *^c^	0.05 *^c^	0.03 ^c^	−0.06 **^c^	0.03 ^c^	0.11 **^c^

Note. Boldfaced numbers on the diagonal represent Cronbach’s alpha; Sex: 0 = male, 1 = female; Age and Tenure in years; M = means; SD = standard deviations; * *p* < 0.05; ** *p* < 0.01. ^a^ = Pearson’s correlation coefficients; ^b^ = Spearman’s rho correlation coefficients; ^c^ = Kendall’s coefficients of rank correlation tau-subb. Total scale = the overall score derived from the 8 items of the HABS-U; Non-physical aggression = the score obtained from the HABS-U sub-dimension that measures non-physical aggression; Physical aggression = the score obtained from the HABS-U sub-dimension that measures physical aggression.

**Table 6:** Mean, standard deviations, *t*-values of aggression scale total scores and its dimensions across gender and roles.

	Men (*n* = 317)		Women (*n* = 1114)		*t*	*p*	95% CI		Cohen’s *d*
	M	SD	M	SD			LL	UL	
Aggression (total score)	0.63	0.70	0.68	0.65	−1.21	0.225	−0.13	0.03	-
Non-physical aggression	0.81	0.88	0.86	0.82	−1.03	0.301	−0.16	0.05	-
Verbal aggression	0.11	0.36	0.14	0.42	−1.53	0.126	−0.09	0.01	-

Note. M = mean, SD = standard deviation, 95% CI = 95% confidence intervals; *t* = *t*-test; LL = lower limit; UL= upper limit. Aggression (total score) = the overall score derived from the 8 items of the HABS-U; Non-physical aggression = the score obtained from the HABS-U sub-dimension that measures non-physical aggression; Physical aggression = the score obtained from the HABS-U sub-dimension that measures physical aggression.

**Table 7:** ANOVA between groups for different years of experience and occupational groups regarding total scores and dimensions of HABS-U.

**Dimension**	**Job Tenure**	**M**	**SD**	**F**	**95% CI**	
					**LL**	**UL**
Aggression (total score)	0–5 years	0.71	0.71	1.61	0.64	0.77
	6–15 years	0.70	0.67		0.63	0.77
	>15 years	0.64	0.63		0.59	0.69
Non-physical aggression	0–5 years	0.89	0.89	1.92	0.81	0.98
	6–15 years	0.88	0.85		0.79	0.97
	>15 years	0.80	0.79		0.74	0.86
Physical aggression	0–5 years	0.14	0.41	0.08	0.81	0.18
	6–15 years	0.14	0.38		0.10	0.18
	>15 years	0.15	0.41		0.10	0.18
**Dimension**	**Role**	**Mean**	**SD**	**F**	**95% CI**	
					**LL**	**UL**
Aggression (total score)	Doctor	0.57	0.48	8.82 ***	0.50	0.63
	Nurse	0.77	0.69		0.71	0.83
	Healthcare assistants	0.61	0.59		0.50	0.71
	Laboratory technician	0.50	0.64		0.39	0.62
	Other healthcare professions	0.56	0.56		0.47	0.64
Non-physical aggression	Doctor	0.73	0.62	6.53 ***	0.65	0.81
	Nurse	0.94	0.85		0.87	1.02
	Healthcare assistants	0.74	0.74		0.61	0.87
	Laboratory technician	0.66	0.84		0.51	0.80
	Other healthcare professions	0.72	0.71		0.61	0.82
Physical aggression	Doctor	0.07	0.25	13.55 ***	0.04	0.30
	Nurse	0.25	0.54		0.20	0.30
	Healthcare assistants	0.22	0.49		0.13	0.30
	Laboratory technician	0.05	0.18		0.01	0.08
	Other healthcare professions	0.06	0.25		0.03	0.10

Note. SD = standard deviations; 95% CI = 95% confidence intervals; LL = lower limit; UL = upper limit; *** *p* < 0.001. Aggression (total score) = the overall score derived from eight items of the HABS-U; Non-physical aggression = the score obtained from the HABS-U sub-dimension that measures non-physical aggression; Physical aggression = the score obtained from the HABS-U sub-dimension that measures physical aggression.

## Data Availability

Data that support the findings of this study and the Italian version of the items are available upon reasonable request from the first author.
